# Salsolinol, an Endogenous Compound Triggers a Two-Phase Opposing Action in the Central Nervous System

**DOI:** 10.1007/s12640-014-9511-y

**Published:** 2014-12-24

**Authors:** Edyta Możdżeń, Małgorzata Kajta, Agnieszka Wąsik, Tomasz Lenda, Lucyna Antkiewicz-Michaluk

**Affiliations:** 1 Department of Neurochemistry, Institute of Pharmacology Polish Academy of Sciences, 12 Smętna Street, 31-343 Kraków, Poland; 2 Department of Neuroendocrinology, Institute of Pharmacology Polish Academy of Sciences, 12 Smętna Street, 31-343 Kraków, Poland; 3 Department of Neuropsychopharmacology, Institute of Pharmacology Polish Academy of Sciences, 12 Smętna Street, 31-343 Kraków, Poland

**Keywords:** Salsolinol, apoptosis, Caspase-3, Lactate dehydrogenase, α-Synuclein, Tyrosine hydroxylase, Dopamine metabolism

## Abstract

Salsolinol (1-methyl-6,7-dihydroxy-1,2,3,4-tetrahydroisoquinoline), an endogenous compound present in the brain, was suspected of participation in the etiopathogenesis of Parkinson’s disease, the most common serious movement disorder worldwide. In this study, we evaluated the effect of different (50, 100, and 500 µM) concentrations of salsolinol on markers of glutamate-induced apoptotic and neurotoxic cell damage, such as caspase-3 activity, lactate dehydrogenase (LDH) release, and the loss of mitochondrial membrane potential. Biochemical data were complemented with the cellular analysis, including Hoechst 33342 and calcein AM staining, to visualize apoptotic DNA-fragmentation and to assess cell survival, respectively. The assessment of all investigated parameters was performed in primary cultures of rat or mouse hippocampal and striatum cells. Our study showed that salsolinol had biphasic effects, namely, at lower concentrations (50 and 100 µM), it demonstrated a distinct neuroprotective activity, whereas in the highest one (500 µM) caused neurotoxic effect. Salsolinol in concentrations of 50 and 100 µM significantly antagonized the pro-apoptotic and neurotoxic effects caused by 1 mM glutamate. Salsolinol diminished the number of bright fragmented nuclei with condensed chromatin and increased cell survival in Hoechst 33342 and calcein AM staining in hippocampal cultures. Additionally, in the low 50 µM concentration, it produced a significant inhibition of glutamate-induced loss of membrane mitochondrial potential. Only the highest concentration of salsolinol (500 µM) enhanced the glutamate excitotoxicity. Ex vivo studies indicated that both acute and chronic administration of salsolinol did not affect the dopamine metabolism, its striatal concentration or α-synuclein and tyrosine hydroxylase protein level in the rat substantia nigra and striatum. Summarizing, the present studies exclude possibility that salsolinol under physiological conditions could be an endogenous factor involved in the neurogenerative processes; conversely, it can exert a protective action on nerve cells in the brain. These findings may have important implications for the development of the new strategies to treat or prevent neural degeneration.

## Introduction

**Fig. 1:**
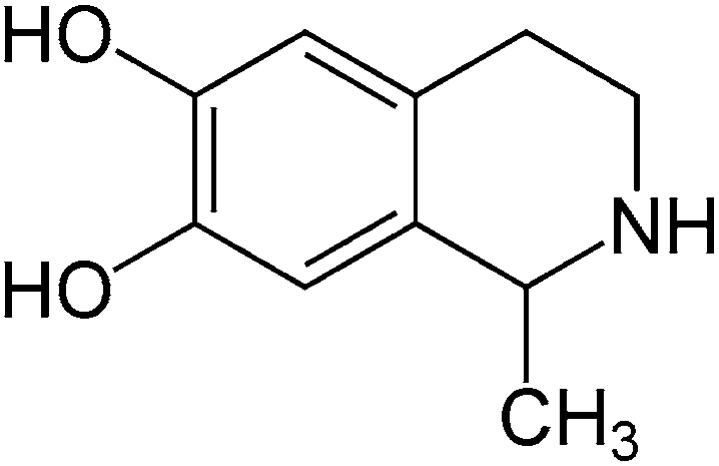
The chemical structure of 1-methyl-6,7-dihydroxy-1,2,3,4-tetrahydroisoquinoline (salsolinol)

Tetrahydroisoquinolines (THIQs) are endogenous substances which have been detected both in the rat and human brain (Deng et al. 1996; Nagatsu 1997; Baum et al. 1999; Haber et al. 1999; Naoi and Maruyama 1999; Musshoff et al. 2000). Because of their structural similarity to 1-methyl-4-phenyl-1,2,3,6-tetrahydropyridine that have been known as a potential causative factor of Parkinson’s disease, tetrahydroisoquinoline alkaloids are considered to be endogenous neurotoxins (Naoi et al. 1994; Maruyama et al. 1997; Nagatsu 1997; Naoi and Maruyama 1999). 1-Methyl-6,7-dihydroxy-1,2,3,4-tetrahydroisoquinoline (salsolinol; its chemical structure is shown in Fig. 1) is one of such compounds. In vitro studies showed that salsolinol and especially its derivative *N*-methyl-salsolinol caused apoptosis of dopamine cells, although the molecular mechanism remains unclear (Akao et al. 1999; Storch et al. 2000; Kim et al. 2001; Naoi et al. 2002).

Salsolinol can be formed in the mammalian brain by three different mechanisms: (1) via the nonenzymatic Pictet–Spengler condensation of dopamine and aldehydes producing salsolinol as two racemic isomers (*R* or *S*); (2) by the nonenzymatic condensation of dopamine and pyruvate yielding 1-carboxyl-tetrahydroisoquinoline, followed by decarboxylation and reduction, which produces (*R*)-salsolinol; (3) by selective synthesis of (*R*)-salsolinol from dopamine and acetaldehyde, the level of which is increased after ethanol intake. Apart from that salsolinol has also been detected in certain beverages and food stuffs, including soy sauce, cheese, chocolate, beef, beer, port wine, and dried bananas (Niwa et al. 1989; Strolin Benedetti et al. 1989; Deng et al. 1997; Cai and Liu 2008).

Salsolinol has been found and demonstrated recently to play an essential physiological role by several groups of scientists pointing to its significant regulatory role in the prolactin release in the neuro-intermediate lobe of the brain (Toth et al. 2001; Homicsko et al. 2003; Naoi et al. 2004; Szekacs et al. 2007; Hashizume et al. 2010; Jin et al. 2014) or the sympathoadrenal system activity (Bodnar et al. 2004).

On the other hand, salsolinol was also found to be involved in neurotoxicity processes altering the normal function and survival of dopamine neurons. The compound caused oxidative stress and acted as an inhibitor of mitochondrial energy supply, and might be responsible for some neurological and psychiatric disorders (Niwa et al. 1993; Nagatsu 1997). Consequently, salsolinol has been proposed to be an endogenous compound participating in the ethiopathogenesis of Parkinson’s disease (Nagatsu 1997; Naoi et al. 1997; Antkiewicz-Michaluk et al. 1998; Lorenc-Koci et al. 2000; Antkiewicz-Michaluk 2002; Mravec 2006). However, it should be noticed that in clinical research carried out on the lumber cerebrospinal fluid (CSF) samples taken from early and advanced parkinsonian patients, salsolinol concentrations were significantly enhanced only in patients with signs of dementia (Antkiewicz-Michaluk et al. 1997).

In that light the question arises whether neurotoxic effect of salsolinol in the brain is connected only with dopaminergic structures. It would be also interesting to examine whether in vitro effects of salsolinol may correspond with the ex vivo experiments.

In order to solve these problems, we investigated in the present study the effect of salsolinol on glutamate-evoked neurotoxicity in the primary cultures of mouse and rat brain. The effects of different salsolinol concentrations on apoptosis markers in in vitro models in rodents and after its in vivo administration on dopamine metabolism in the rat striatum and substantia nigra were assessed.

## Materials and Methods

### Animals and Treatment

The ex vivo experiments were carried out on male Wistar rats with initial body weight of 220–240 g. The animals were kept under standard laboratory conditions with free access to standard laboratory food and tap water, at room temperature of 22 °C with an artificial day cycle (12/12 h, light on at 7 a.m.).

The rats were administrated salsolinol at a dose of 100 mg/kg intraperitoneally (i.p.) once or chronically for 14 consecutive days. Control rats were treated with an appropriate solvent. Animals were killed by decapitation 2 or 24 and 3 or 24 h after the last drug injection to assay the level of dopamine and its metabolites, and α-synuclein and tyrosine hydroxylase, respectively. To perform the above analysis, the substantia nigra and striatum were isolated from the brain. All experiments were performed between 9.00 a.m. and 4.00 p.m.

All the procedures were carried out in accordance with the National Institutes of Health Guide for the Care and Use of Laboratory Animals and were granted an approval from the Bioethics Commission as compliant with Polish Law. All the experimental procedures were approved by the Local Bioethics Commission of the Institute of Pharmacology, Polish Academy of Sciences in Cracow.

### Drugs

Salsolinol (1-methyl-6,7-dihydroxy-1,2,3,4-tetrahydroisoquinoline; Sigma-Aldrich) was dissolved in 0.9 % NaCl solution. Glutamic acid was obtained from Sigma-Aldrich (St. Louis, MO, USA), whereas Hoechst 33342 and calcein AM were purchased from Molecular Probes (Eugene, OR, USA). The chemical structure of the salsolinol is shown in Fig. 1.

### Primary Hippocampal and Striatum Cell Cultures

Hippocampal and striatum tissues for primary cultures were collected from Wistar rat or Swiss mouse embryos (Charles River, Germany) on 15th–17th day of gestation and were cultured essentially as described earlier (Kajta et al. 2007, 2009a). Animal care followed official governmental guidelines, and all efforts were made to minimize the number of animals used and their suffering. All procedures were carried out in accordance with the National Institutes of Health Guidelines for the Care and Use of Laboratory Animals and were approved by the Bioethics Commission as being compliant with Polish Law (21 August 1997). The cells were suspended in estrogen-free neurobasal medium supplemented with B27 and plated at a density of 2.5 × 10^5^ cell/cm^2^ onto poly-ornithine-coated (0.01 mg per ml) multi-well plates. The cultures were maintained at 37 °C in a humidified atmosphere containing 5 % CO_2_ for 7 days in vitro (DIV) prior to experimentation. The level of astrocytes in cell cultures, as determined by the content of intermediate filament protein glial fibrillary acidic protein (GFAP), did not exceed 10 % (Kajta et al. 2004, 2014).

#### Treatment

Primary hippocampal and striatum cell cultures were exposed to glutamic acid (1 mM), salsolinol (50, 100, 500 µM) or glutamic acid, and salsolinol for 24 h. To avoid unspecific effects in our study, salsolinol was used in the concentrations which did not affect the control level of caspase-3 activity and LDH release. All the compounds were originally dissolved in dimethyl sulfoxide (DMSO) and then further diluted in culture medium so that DMSO concentrations remained below 0.1 %.

#### Identification of Apoptotic Cells

In order to visually assess apoptotic changes in hippocampal cells, Hoechst 33342-staining was applied 24 h after initial treatment, as described previously (Kajta et al. 2007, 2013). Before dye application, hippocampal cells cultured on glass cover slips were washed with 10 mM phosphate-buffered saline (PBS) and exposed to Hoechst 33342 (0.6 mg/ml), at room temperature (RT) for 5 min. Cells with bright blue fragmented nuclei showing condensed chromatin were identified as apoptotic cells. Qualitative analysis was performed using a fluorescence microscope (Leica Microsystems Wetzlar GmbH, Wetzlar, Germany) connected to a CoolSnap camera (Vision Systems GmbH, Puchheim, Germany) with the use of MetaMorph Software.

We counted fragmented nuclei and presented them as a percentage of the vehicle-treated control. The total number of nuclei in each experimental group ranged between 480 and 530. At least three slides were made from three independent culture platings.

#### Staining with Calcein AM

Staining with calcein AM was used to measure intracellular esterase activity in hippocampal cultures 24 h after initial treatment (Kajta et al. 2007). To block esterase activity present in the growth media, cells were washed with PBS. The cells grown on glass cover slips were then incubated in 2 µM calcein AM in PBS at RT for 10 min. Cells with bright yellow cytoplasm were identified as live cells. A fluorescence microscope (Leica Microsystems Wetzlar GmbH, Wetzlar, Germany) connected to a CoolSnap camera (Vision Systems GmbH, Puchheim, Germany) with MetaMorph software was used for qualitative analyses.

#### Assessment of Mitochondrial Membrane Potential

The mitochondrial membrane potential was evaluated with the JC-1 Assay Kit, which utilizes a cationic dye 5,5′,6,6′-tetrachloro-1,1′,3,3′-tetraethylbenzimidazolylcarbo-cyanine iodide. In healthy cells, the dye aggregates and stains the mitochondria bright red, whereas in apoptotic cells, the mitochondrial cytoplasm contains a green fluorescent monomeric form (Hirsch et al. 1998). The assessment of loss of mitochondrial membrane potential, which is a hallmark of apoptosis, was performed in the hippocampal cultures treated for 6 h with glutamic acid alone or in combination with salsolinol (50, 500 µM). The cells were incubated with JC-1 solution for 25 min, and red (550/600 nm) and green (485/535 nm) fluorescences were measured with an Infinite M1000 microplate reader (Tecan, Austria). The data were analyzed with I-control software, normalized to the fluorescence in vehicle-treated cells, and expressed as the mean red to green fluorescence ratio ± SEM of three to four independence experiments. The fluorescence of blanks, i.e., no-enzyme controls, was subtracted from each value.

#### Assessment of Caspase-3 Activity

Caspase-3 activity was assayed according to Nicholson et al. (1995), in samples treated for 24 h with glutamic acid (1 mM) alone or in combination with the test compound. The assessment of caspase-3 activity was performed as previously described (Kajta et al. 2009b). Cell lysates were incubated at 36 °C with a colorimetric substrate preferentially cleaved by caspase-3: Ac-DEVD-pNA (*N*-acetyl-asp-glu-val-asp-*p*-nitro-anilide). The levels of *p*-nitroanilide were monitored continuously over 60 min with a Multiskan Spectrum Microplate Spectrophotometer (Thermo Labsystems, Vantaa, Finland). Data were analyzed with Ascent Software, normalized to the absorbance in vehicle-treated cells, and expressed as the mean percentage of control ± SEM of three to four independent experiments. Absorbance of blanks, i.e., no-enzyme controls, was subtracted from each value.

#### Measurement of Lactate Dehydrogenase Activity

In order to estimate cell death, the level of lactate dehydrogenase (LDH) released from damaged cells into culture media was measured 24 h after treatment with glutamic acid (1 mM) and salsolinol (50, 100, and 500 µM). LDH release was measured as previously described (Kajta et al. 2004). Cell-free culture supernatants were collected from each well and incubated with the appropriate reagent mixture according to the manufacturer’s instructions (Cytotoxicity Detection Kit) at RT for 30–60 min depending on the reaction progress. The intensity of red color formed in the assay mixture and measured at a wavelength of 490 nm was proportional to LDH activity and to the number of damaged cells. Data were normalized to the activity of LDH released from vehicle-treated cells (100 %) and expressed as the mean percent of the control of three to four independent experiments. The total LDH release was determined in the cell cultures treated with 1 % Triton X-100 for 24 h. The total LDH release reached a value of 1.108 ± 0.154 U/h/100 µg protein. In control cultures, the absolute value of LDH activity was 0.270 ± 0.011 U/h/100 µg protein and was similar to that obtained by Mytilineou et al. (1998).

### HPLC Analysis of the Concentration of Dopamine and Its Metabolites

The animals were killed by decapitation 2 or 24 h after the last salsolinol (100 mg/kg i.p.) injection. The brains were rapidly removed and dissected on an ice-cold glass plate. The substantia nigra and striatum were isolated and immediately frozen on solid CO_2_ (−80 °C) until used for biochemical assay. Dopamine (DA) and its metabolites, the intraneuronal, 3,4-dihydroxyphenylacetic acid (DOPAC); the extraneuronal, 3-methoxytyramine (3-MT) and the final metabolite; and homovanillic acid (HVA) were assayed by means of high-performance liquid chromatography (HPLC) with electrochemical detection (ED). The tissue samples were weighted and homogenized in ice-cold 0.1 M trichloroacetic acid containing 0.05 mM ascorbic acid. After centrifugation (10,000×*g*, 5 min), the supernatants were filtered through RC 58 0.2 µm cellulose membranes (Bioanalytical Systems, West Lafayette, IN, USA). The chromatograph HP 1050 (Hewlett-Packard, Golden, CO, USA) was equipped with Hypersil columns BDS-C18 (4 × 100 mm, 3 µm). The mobile phase consisted of 0.05 M citrate–phosphate buffer, pH 3.5; 0.1 mM EDTA; 1 mM sodium octyl sulfonate; and 3.5 % methanol. The flow rate was maintained at 1 ml/min. Dopamine and their metabolites were quantified by peak area comparisons with standards run on the day of analysis (ChemStation, Hewlett-Packard software computer program).

### Western Blot Analysis of α-synuclein and Tyrosine Hydroxylase Protein

After dissection, the substantia nigra was immediately frozen on dry ice and stored at −80 °C until analysis. The tissue was homogenized on ice in 20 volumes of RIPA buffer (150 mM NaCl, 1 % NP-40, 0.5 % sodium deoxycholate, 0.1 % SDS, 50 mM Tris, pH 8.0) containing a mixture of protease inhibitors (Pierce). Protein concentration in the supernatants was determined using bicinchoninic acid protein assay kit (Pierce). Afterwards, the samples containing 5 μg of total protein were fractionated by 10 % sodium dodecyl sulfate-polyacrylamide gel electrophoresis (SDS-PAGE), according to Laemnili (1970), and processed in order to detect α-synuclein or tyrosine hydroxylase (TH). Proteins from the resolved gels were then transferred to nitrocellulose membranes (Sigma). Nonspecific binding sites were blocked overnight at 4 °C by a 3 % BSA in Tris-buffered saline with 0.5 % Tween 20 (TBS-T) and incubated for 2 h with a mouse monoclonal anti-α-synuclein antibody (BD Transduction Laboratories, dilution 1:2000) or mouse monoclonal anti-TH antibody (Millipore, dilution 1:4000) in 1 % BSA at RT. After three subsequent washes in TBS-T, membranes were processed according to the standard BM Chemiluminescence Western Blotting Kit protocol (Roche Applied Science). Following immunoblot visualization, membranes were blocked with 5 % non-fat dry milk in TBS for 10 min at RT and dried on absorbent filter paper. Afterwards, blots were erased in 62.5 mM Tris pH 6.8, 2 % SDS, 100 mM 2-mercaptoethanol for 30 min at 50 °C, washed twice with TBS, and blocked overnight with 5 % non-fat dry milk in TBS at 4 °C. As a control for level normalization, the erased blots were processed with mouse monoclonal antiβ-actin antibody (Santa Cruz Biotechnology, Inc., dilution 1:10000), as described above.

The amounts of protein per lane as well as antibody concentrations were optimized in pilot studies so that threefold differences in protein content were linearly reflected on the immunoblots.

The signals were visualized and quantified by the densitometric analysis with the FUJI-LAS 4000 system and Fuji Multi Gage Software. The results are presented as a percentage of the control of the analyzed protein: β-actin ratio ± SEM.

### Data Analysis

Statistical tests were performed on raw data expressed as the mean arbitrary absorbance or fluorescence units per well containing 50,000 cells (measurements of caspase-3, LDH, mitochondrial potential). A one-way analysis of variance (ANOVA) was used to determine overall significance. Differences between control and experimental groups were assessed with a post hoc Newman–Keuls test. Significant differences were marked as follows: *P < 0.05, **P < 0.01, ***P < 0.001 (versus control group); ^#^ *P* < 0.05, ^##^ *P* < 0.01, ^###^ *P* < 0.001 (versus glutamic acid group).

Following Shapiro–Wilk tests, for normality, the data of neurochemical studies were compared using Student’s *t* test for independent groups (control versus salsolinol 100 mg/kg group). The null hypothesis of the lack of differences between the investigated groups was adopted (*P* > 0.05). The alternative hypothesis commanded the existence of differences between groups.

The total DA catabolism rate was assessed from the ratio of the final DA metabolite concentration, HVA to DA concentration and expressed as the catabolic rate index (HVA)/(DA) × 100; the rate of DA MAO-dependent oxidation as the ratio: (DOPAC)/(DA) × 100; the rate of DA COMT-dependent *O*-methylation as the ratio: (3-MT)/(DA) × 100; and the factor of DA re-uptake inhibition as the ratio: (3-MT)/(DOPAC) × 100. The indices were calculated using concentrations from individual tissue samples (Antkiewicz-Michaluk et al. 2001).

Statistical significance of the data from Western blot analysis was assessed using a one-way analysis of variance (ANOVA) followed (if significant) by Tukey test for post hoc comparison with the control (saline) group.

## Results

### The Effects of Salsolinol on Glutamate-Induced Caspase-3 Activity and LDH Release in Rat Hippocampal and Mouse Striatum Cultures

#### Rat Hippocampal Cultures

**Fig. 2:**
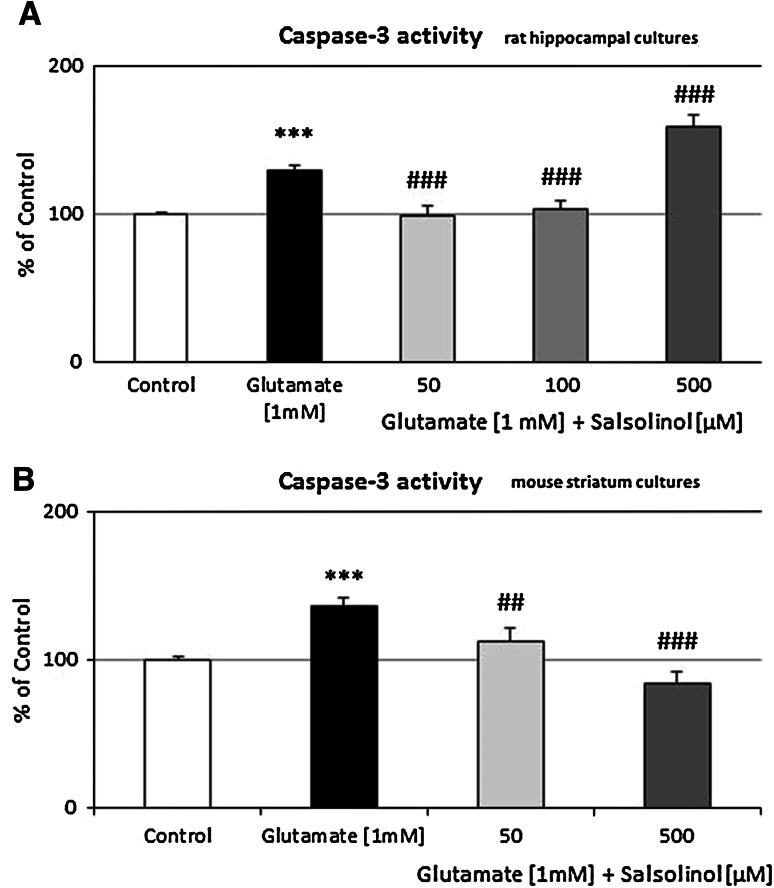
The effect of salsolinol on glutamate-induced caspase-3 activity in **a** rat hippocampal and **b** mouse striatum cultures. Cells were treated either with glutamic acid (1 mM) or salsolinol (50, 100, and 500 µM) alone or in combination. The results are presented as a percentage of control. Each bar represents the mean of three or four independent experiments ± SEM. The number of replicantes is each experiment ranged from 5 to 8. ***P < 0.001 versus control cultures; ^###^ *P* < 0.001 versus cultures exposed to glutamic acid

In hippocampal cultures exposed to 1 mM glutamic acid for 24 h, the activity of caspase-3 increased by 30 % (*P* < 0.01) (Fig. 2a). In the presence of glutamate and salsolinol in the low concentrations (50 and 100 µM), the activity of caspase-3 was diminished by amount 20 % compared to the glutamate-induced value (*P* < 0.001). In contrast, the highest dose of salsolinol (500 µM) significantly intensified glutamate-activated caspase-3 (up to 125 %, *P* < 0.001) (Fig. 2a).

**Fig. 3:**
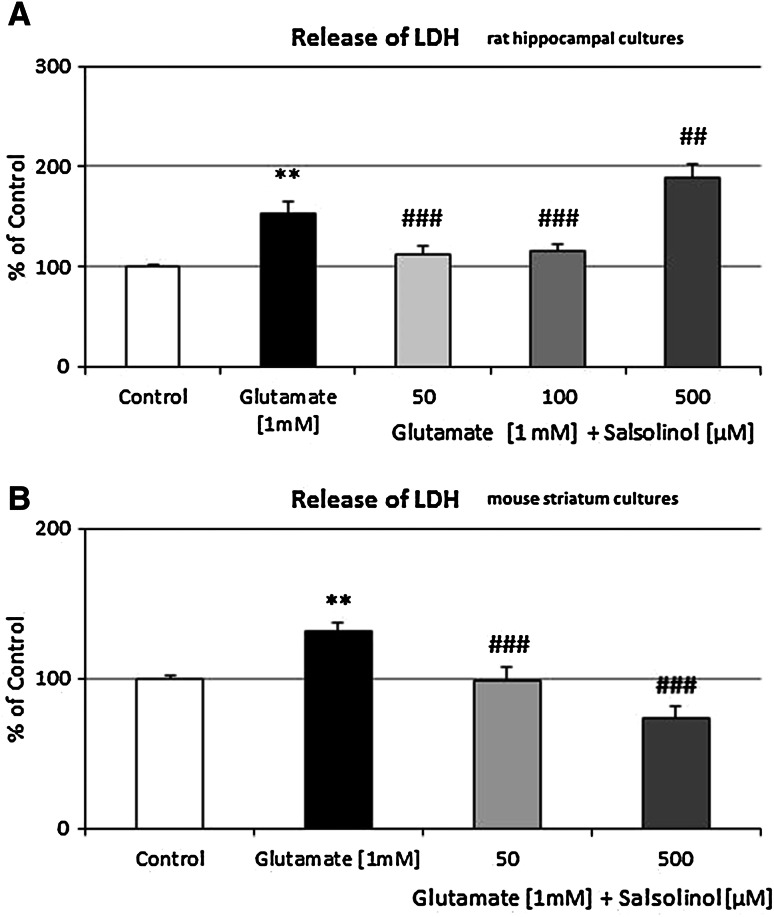
The effect of salsolinol on glutamate-induced release of LDH in **a** rat hippocampal and **b** mouse striatum cultures. Cells were treated either with glutamate (1 mM) alone or in combination with salsolinol (50, 100, or 500 µM). The results are presented as a percentage of control. Each bar represents the mean of three to four independent experiments ± SEM. The number of replicantes in each experiment ranged from 5 to 8. **P < 0.01 versus control cultures; ^#^ *P* < 0.05, ^###^ *P* < 0.001 versus the cultures exposed to glutamic acid

LDH release increased with the duration of glutamic acid treatment by 54 % (*P* < 0.01) (Fig. 3a). Salsolinol was effective in the lower concentrations (50 and 100 µM)—it inhibited glutamate-induced LDH release by amount 25–27 % (*P* < 0.001). After 24 h of treatment, salsolinol increased glutamate-induced LDH release only at the highest concentration of 500 µM (up to 123 %, *P* < 0.01) (Fig. 3a).

#### Mouse Striatum Cultures

In striatum cultures exposed to 1 mM glutamic acid for 24 h, the activity of caspase-3 increased by 40 % (*P* < 0.01) (Fig. 2b). Salsolinol administration in both concentrations 50 and 500 µM inhibited the activity of caspase-3 by amount 17 % and 38 %, respectively (compared to the glutamic acid effects, *P* < 0.001) (Fig. 2b).

LDH release increased with the duration of glutamic acid treatment by 33 % (*P* < 0.01) (Fig. 3b). Both concentrations of salsolinol (low—50 µM, the highest—500 µM) effectively inhibited glutamate-induced LDH release by amount 33 and 44 % (*P* < 0.001) (Fig. 3b), respectively.

### The Effects of Salsolinol on Glutamate-Induced Changes in Calcein AM and Hoechst 33342 Staining in Hippocampal Cultures

**Fig. 4:**
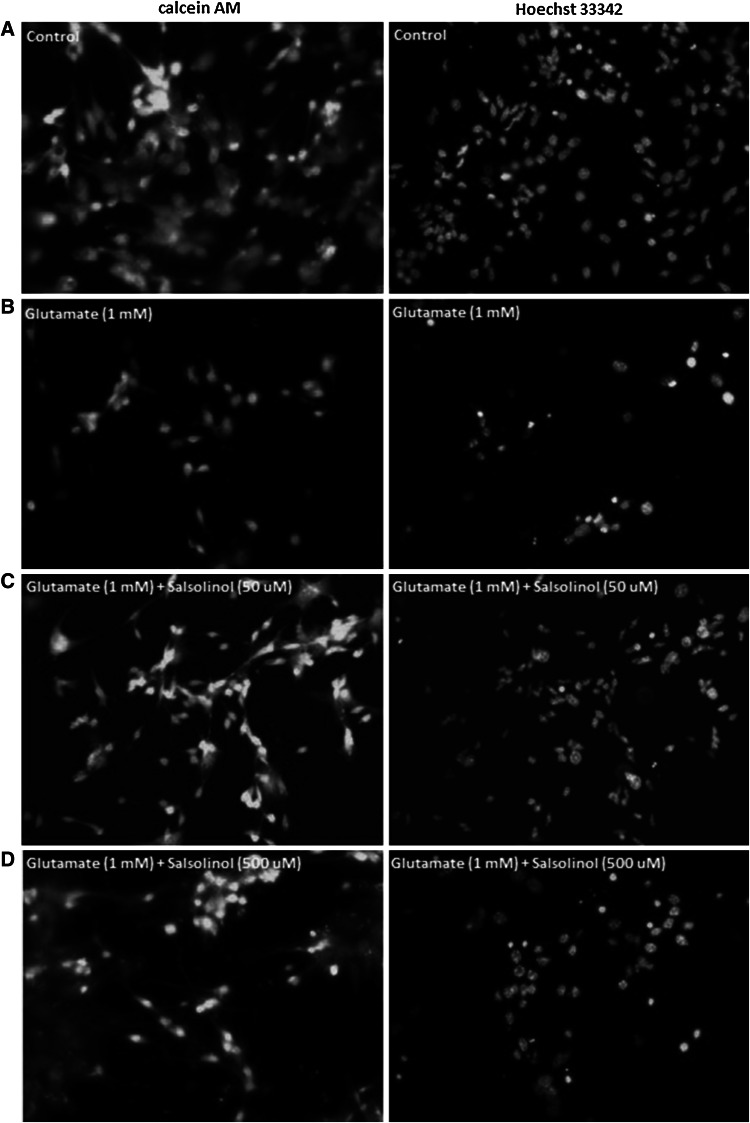
The effect of salsolinol (50 or 500 µM) on glutamate-induced (1 mM) changes in calcein AM (*first column*) and Hoechst 33342 (*second column*) staining in rat hippocampal cultures at seven DIV, examined 24 h post-treatment. **a** Control, **b** glutamate (1 mM), **c** glutamate (1 mM) + salsolinol (50 µM), **d** glutamate (1 mM) + Salsolinol (500 µM). Cells were cultured on glass cover slips, washed with 10 mM PBS, and exposed to 2 µM calcein AM at RT for 10 min. Cells were then rewashed and incubated with Hoechst 33342 (0.6 µg/ml) at RT for 5 min. Cells with bright fragmented nuclei showing condensed chromatin were identified as undergoing apoptosis, whereas cells with light-colored cytoplasm were identified as living cells. Hoechst 33342 stain is one of the most common used parts of a family of blue fluorescent dyes used to stain DNA. (For interpretation of the references to *color* in this figure legend, the reader is referred to the web version of this article)

**Fig. 5:**
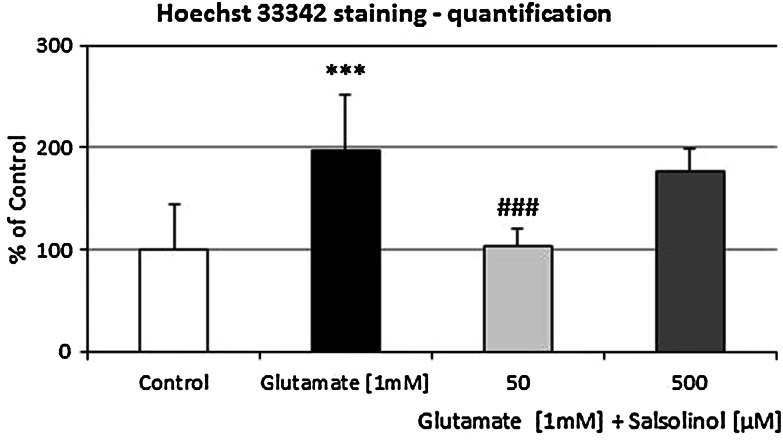
The effect of salsolinol (50 or 500 µM) on glutamate-induced (1 mM) changes in Hoechst 33342 staining in rat hippocampal cultures—quantification. Fragmented nuclei were counted and presented as a percentage of the vehicle-treated control. The total number of nuclei in each experimental group ranged between 480 and 530. At least three slides were made from three independent culture platings

A continuous 24 h exposure of hippocampal cultures to glutamic acid (1 mM) diminished the density of living cells on seven DIV, as indicated by the decreased number of cells with light-colored cytoplasm (Fig. 4b). Treatment with glutamic acid substantially enhanced the number of bright fragmented nuclei with condensed chromatin (by amount 97 % vs. control group; *P* < 0.001), which is typical of cells undergoing apoptosis (Figs. 4b, 5).

Co-treatment with salsolinol (50 µM) normalized the number of healthy living cells and diminished the number of fragmented nuclei (*P* < 0.001) (Figs. 4c, 5). In contrast to this, co-treatment with the highest dose of salsolinol (500 µM) enhanced the glutamate-induced changes in Hoechst 33342 and calcein AM staining in the hippocampal cultures (Figs. 4d, 5).

### The Effects of Salsolinol on Glutamate-Induced Loss of Mitochondrial Membrane Potential in Mouse Hippocampal Cultures

**Fig. 6:**
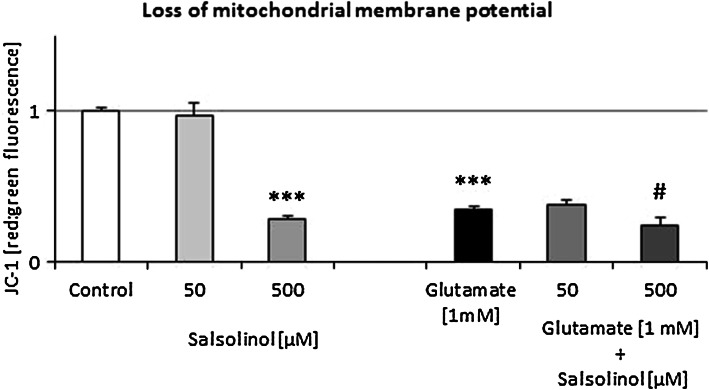
The effect of salsolinol on glutamate-induced loss of mitochondrial membrane potential in mouse hippocampal cultures. Primary hippocampal cultures were treated with glutamic acid (1 mM) or salsolinol (50 or 500 µM) alone or in combination, for 6 h. The mitochondrial membrane potential was detected with JC-1 Assay Kit. The results were normalized to the fluorescence in vehicle-treated cells and expressed as red to green fluorescence ratio. Each bar represents the mean of three to four independent experiments ± SEM. The number of replicantes in each experimental ranged from 5 to 8. ***P < 0.001 versus control cultures; ^#^ *P* < 0.05, ^##^ *P* < 0.01 versus the cultures exposed to glutamic acid

Six-hour exposure of hippocampal cultures to glutamic acid (1 mM) caused over 66 % loss of the mitochondrial membrane potential in the neuronal cells (*P* < 0.001) (Fig. 6). Salsolinol used in a low concentration (50 µM) in primary hippocampal cultures did not affect the mitochondrial membrane potential but produced two-phase effect on glutamate-induced toxicity. The highest dose of salsolinol (500 µM) produced the effect similar to glutamate (*P* < 0.001). Salsolinol in investigated concentration did not cause statistically significant changes in the effect of glutamate on the loss of mitochondrial membrane potential (Fig. 6).

### The Effects of Salsolinol on Dopamine, Its Metabolites Concentration in Rats, and the Rate of Dopamine Metabolism in Substantia Nigra and Striatum (Tables 1, 2)

#### Substantia Nigra

The statistical analysis showed no effect of acute administration of salsolinol on dopamine and its metabolites concentrations, and the rate of dopamine metabolism in substantia nigra (Table 1a).

**Table 1:** The effect of salsolinol on dopamine, its metabolites concentration in rats, and the rate of dopamine metabolism in substantia nigra

Treatment	DA	DOPAC	3-MT	HVA	HVA/DA	DOPAC/DA	3-MT/DA
a: Salsolinol—acute							
Control	889 ± 125	212 ± 84	49 ± 11	106 ± 37	12 ± 4	24 ± 11	6 ± 1
Salsolinol	773 ± 100	191 ± 38	79 ± 52	94 ± 21	14 ± 3	24 ± 3	7 ± 2
*t*	1.77	0.64	−1.36	0.78	−1.20	0.15	−1.83
df	12	12	12	12	12	12	12
*P*	N.S.	N.S.	N.S.	N.S.	N.S.	N.S.	N.S.
b: Salsolinol—chronic, 2h withdrawal							
Control	845 ± 162	215 ± 49	74 ± 18	152 ± 30	19 ± 5	25 ± 2	9 ± 1
Salsolinol	913 ± 156	198 ± 37	64 ± 9	147 ± 39	16 ± 3	22 ± 3*	7 ± 2
*t*	−0.80	0.74	1.33	0.27	1.11	2.62	2.07
df	12	12	12	12	12	12	12
*P*	N.S.	N.S.	N.S.	N.S.	N.S.	*P* < 0.05	N.S.
c: Salsolinol—chronic, 24h withdrawal							
Control	795 ± 250	212 ± 48	72 ± 18	150 ± 36	20 ± 6	27 ± 4	10 ± 4
Salsolinol	675 ± 126	191 ± 38	60 ± 12	104 ± 44*	16 ± 7	29 ± 5	9 ± 3
*t*	1.13	0.88	1.46	2.18	1.24	−0.62	0.21
df	12	12	12	12	12	12	12
*P*	N.S.	N.S.	N.S.	*P* ≤ 0.05	N.S.	N.S.	N.S.

Control group received saline. Salsolinol was administrated in a dose 100 mg/kg i.p. once (acute) or for 14 consecutive days (chronic); *N* = 6–8 animals per group. The concentrations of dopamine and its metabolites were measured in ng/g wet tissue. The data are the mean ± SD. Following Shapiro–Wilk test, for normality, the data were compared using Student’s *t* test for independent group (control vs. salsolinol 100 mg/kg group). The null hypothesis of the lack of differences between the investigated groups was adopted (*P* > 0.05). The alternative hypothesis commanded the existence of differences between groups; statistical significance: *  *P* < 0.05, **  *P* < 0.01

**Table 2:** The effect of salsolinol on dopamine, its metabolites concentration in rats, and the rate of dopamine metabolism in striatum

Treatment	DA	DOPAC	3-MT	HVA	HVA/DA	DOPAC/DA	3-MT/DA
a: Salsolinol—acute							
Control	9,042 ± 1,054	1,238 ± 62	438 ± 48	675 ± 113	7.4 ± 0.7	14 ± 2	4.8 ± 0.2
Salsolinol	9,457 ± 1,008	1,584 ± 227**	689 ± 384	897 ± 281	11 ± 3**	16 ± 2*	5.1 ± 0.5
*t *	−0.70	−3.60	−1.57	−1.82	−6.28	−2.32	−1.02
df	10	12	12	12	12	12	12
*P*	N.S.	*P* ≤ 0.01	N.S.	N.S.	*P* ≤ 0.01	*P* < 0.05	N.S.
b: Salsolinol—chronic, 2h withdrawal							
Control	10,439 ± 815	1467 ± 131	554 ± 58	1,149 ± 83	11 ± 1.3	14 ± 2	5.3 ± 0.5
Salsolinol	11,415 ± 1044	1,789 ± 327*	514 ± 26	1,377 ± 296	12 ± 2.1	16 ± 2	4.5 ± 0.5*
*t *	−1.95	−2.42	1.65	−1.96	−1.03	−1.52	2.77
df	12	12	12	12	12	12	12
*P*	N.S.	*P* < 0.05	N.S.	N.S.	N.S.	N.S.	*P* < 0.05
c: Salsolinol—chronic, 24h withdrawal							
Control	13,843 ± 1,626	1,728 ± 178	665 ± 62	1,263 ± 179	9 ± 2	13 ± 1	4.8 ± 0.3
Salsolinol	13,808 ± 1,222	1,719 ± 312	679 ± 127	1,169 ± 405	8 ± 2	12 ± 1	4.9 ± 0.8
t	0.05	0.06	−0.26	0.56	0.75	0.25	−0.30
df	12	12	12	12	12	12	12
*P*	N.S.	N.S.	N.S.	N.S.	N.S.	N.S.	N.S.

Control group received saline. Salsolinol was administrated in a dose 100 mg/kg i.p. once (acute) or for 14 consecutive days (chronic); *N* = 6–8 animals per group. The concentrations of dopamine and its metabolites were measured in ng/g wet tissue. The data are the mean ± SD. Following Shapiro–Wilk test, for normality, the data were compared using Student’s *t* test for independent group (control vs. salsolinol 100 mg/kg group). The null hypothesis of the lack of differences between the investigated groups was adopted (*P* > 0.05). The alternative hypothesis commanded the existence of differences between groups; statistical significance: * *P* < 0.05, ** *P* < 0.01

2 h withdrawal after chronic administration revealed no differences in dopamine and its metabolites levels between investigated groups. Only dopamine catabolism (MAO-dependent oxidation) DOPAC/DA was slightly inhibited (*P* < 0.05) (Table 1b).

After 24 h withdrawal, a slight inhibition of the HVA level was observed (*P* ≤ 0.05) in substantia nigra. The concentration of dopamine and the rate of its metabolism were not changed (Table 1c).

#### Striatum

The statistical analysis revealed no changes in dopamine levels in striatum after acute and chronic (2 and 24 h withdrawal) salsolinol administration (Table 2a–c). Statistically significant elevation in DOPAC concentration was observed after acute (*P* < 0.01) and chronic (2 h withdrawal) salsolinol injection (Table 2a, b).

Acute administration of salsolinol slightly increased the rate of final dopamine metabolism HVA/DA (*P* ≤ 0.01) and dopamine catabolism (MAO-dependent oxidation) DOPAC/DA (*P* < 0.05).

Additionally, 2 h withdrawal after chronic administration of salsolinol slightly inhibited dopamine catabolism (COMT-dependent *O*-methylation) 3-MT/DA (Table 2b). No more changes in dopamine, its metabolites concentrations, and the rate of dopamine metabolism were observed after acute and chronic administration of salsolinol in striatum.

### The Effects of Acute and Chronic Salsolinol Administration on the α-synuclein Level in Rat Substantia Nigra as Measured 3 and 24 h After the Last Dose. Ex Vivo Study

**Fig. 7:**
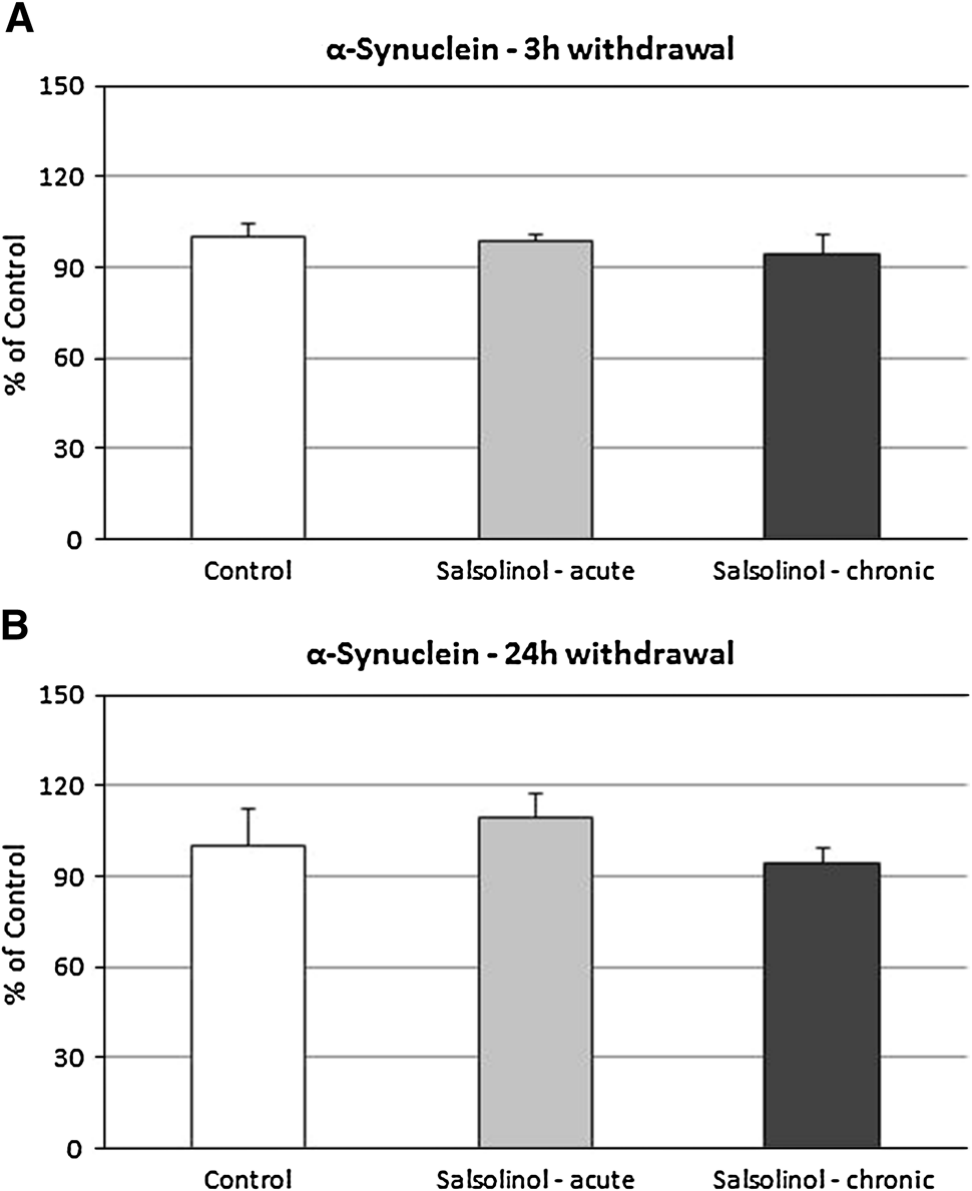
The effects of acute and chronic salsolinol administration on the α-synuclein level in the substantia nigra, **a** 3 h and **b** 24 h withdrawal. Salsolinol was administrated acute or chronic at dose 100 mg/kg i.p. during 14 consecutive days. The control group was treated with saline. The rats were decapitated 3 or 24 h after last injection, respectively. The results are expressed as the mean ± SEM of six samples (*n* = 6 animals per group). Data were analyzed by means of one-way ANOVA followed by Tukey test. Statistical significance: *P < 0.05; **P < 0.01 versus control group

Both acute and chronic (14 consecutive days) administrations of salsolinol (100 mg/kg i.p.) did not change the level of α-synuclein in rats substantia nigra measured 3 or 24 h after the last dose (Fig. 7a, b).

### The Effects of Acute and Chronic Salsolinol Administration on the Tyrosine Hydroxylase Level in Rat Substantia Nigra as Measured 3 and 24 h After the Last Dose. Ex Vivo Study

**Fig. 8:**
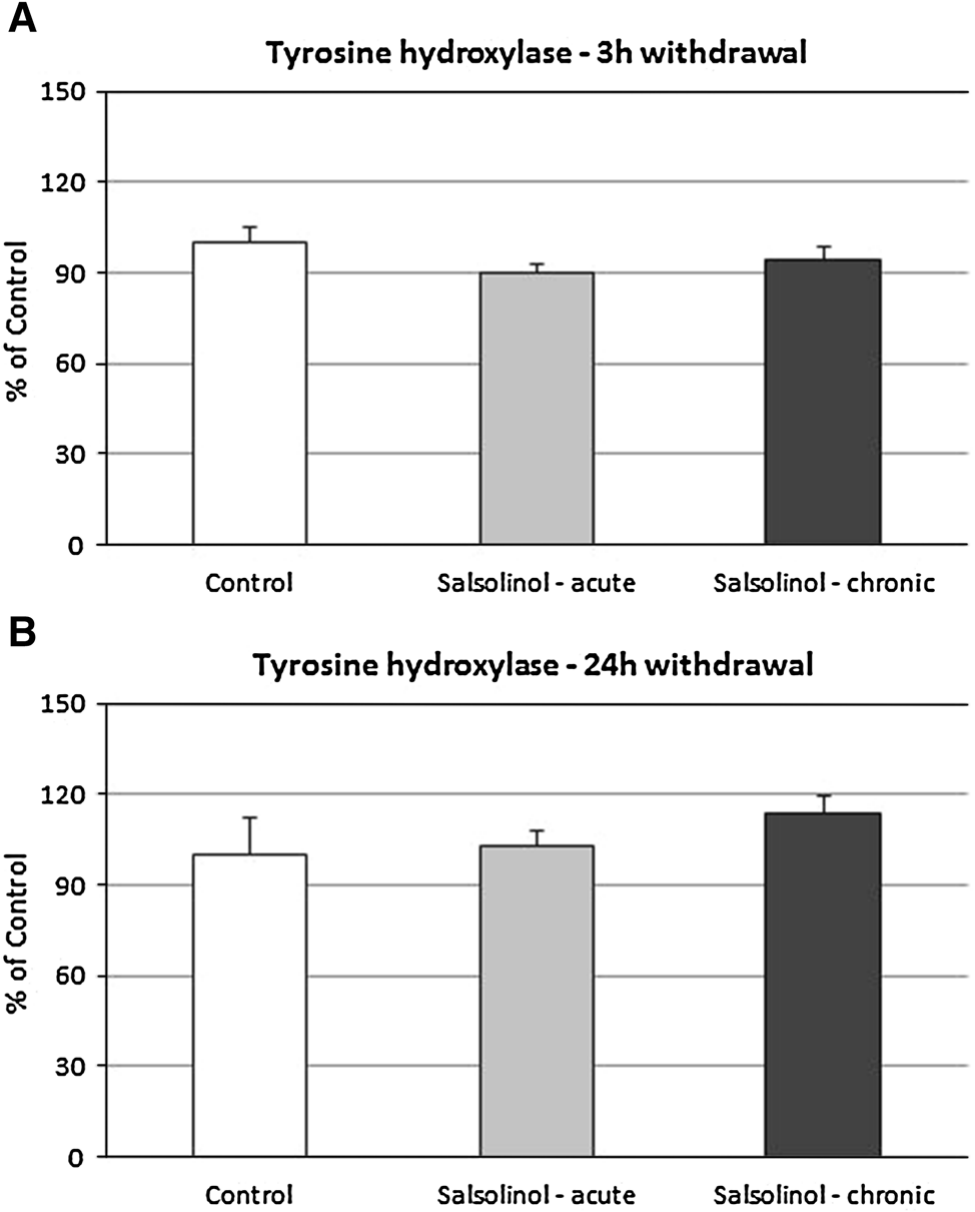
The effects of acute and chronic salsolinol administration on the tyrosine hydroxylase level in the substantia nigra, **a** 3 and **b** 24 h withdrawal. Salsolinol was administrated acute or chronic at dose 100 mg/kg i.p. during 14 consecutive days. The control group was treated with saline. The rats were decapitated 3 or 24 h after last injection, respectively. The results are expressed as the mean ± SEM of six samples (*n* = 6 animals per group). Data were analyzed by means of one-way ANOVA followed by Tukey test. Statistical significance: *P < 0.05; **P < 0.01 versus control group

Both acute and chronic (14 consecutive days) administrations of salsolinol (100 mg/kg i.p.) did not change the level of tyrosine hydroxylase in rats substantia nigra measured 3 or 24 h after the last dose (Fig. 8a, b).

## Discussion

The main finding of this paper is that salsolinol produced concentration-dependent opposing effects on apoptosis markers assessed in in vitro studies in the primary hippocampal cultures of mice and rats. Salsolinol in the low investigated concentrations (50 and 100 µM) possessed neuroprotective activity; however, a tenfold increase in its concentration (500 µM) seemed to be neurotoxic, and significantly intensified glutamate-induced neurotoxicity. Estimation of the levels of two proteins: α-synuclein and tyrosine hydroxylase after chronic in vivo administration of salsolinol did not reveal its neurotoxic effects on dopamine neurons in the substantia nigra. Similarly to this, ex vivo studies did not show any significant adverse effects of salsolinol on the levels of dopamine and its metabolites in the nigrostriatal system: substantia nigra and striatum.

Salsolinol, a catechol isoquinoline, has invited considerable attention due to its structural similarity to dopaminergic neurotoxins, like MPTP. Its high endogenous level in the brain of parkinsonian patients might suggest its possible association with the disease process (Nagatsu and Yoshida 1988; Kotake et al. 1995; Antkiewicz-Michaluk et al. 2001). Salsolinol is considered to be synthesized through a condensation reaction between dopamine and acetaldehyde in the human and mammalian brain. A low concentration of salsolinol was detected in normal human cerebrospinal fluid (Moser and Kompf 1992), brain and urine (Dostert et al. 1989). In contrast, both parkinsonian patients treated with l-DOPA and chronic alcoholics showed a significant elevation in the concentration of salsolinol in CSF and urine (Cohen and Collins 1970; Sandler et al. 1973; Collins et al. 1979; Moser and Kompf 1992). Besides endogenous formation, salsolinol has also been detected in certain beverages and food stuffs, including soy sauce, chocolate, beer, port wine, and dried bananas.

The ability of salsolinol to cross the blood–brain barrier (BBB) has been the subject of interest of several research groups, although, unfortunately, the results obtained so far are not conclusive. Initially, it was commonly thought that salsolinol as a catecholamine and its analogs were not able to migrate from blood into the brain, despite the fact that contradictory results were reported (Origitano et al. 1981; Sjöquist and Magnuson 1980). Several authors have reported that systemically administrated salsolinol is capable of altering behavior (Naoi et al. 1996; Antkiewicz-Michaluk et al. 2000; Matsuzawa et al. 2000; Vetulani et al. 2001), which indirectly suggests that salsolinol could cross the BBB.

A number of in vitro studies have demonstrated that glutamate is a potent neurotoxin capable of destroying neurons by apoptosis (Froissard and Duval 1994; Kajta et al. 2007; 2013; Wąsik et al. 2014). For this reason, the glutamate model of neurotoxicity was used in the presented paper as a good model to study programmed cells death. As investigated so far, exposure to glutamic acid has been associated with an increase in cytosolic Ca^2+^ in the cells (Atlante et al. 2001), and a long-time exposure to glutamate resulted in permanent damage of mitochondria, which occurred simultaneously with a high mitochondrial ROS production (Beal et al. 1997). As presented in this paper, glutamic acid (1 mM) significantly increased the caspase-3 activity (Fig. 2a, b). In rat hippocampal cultures, the lower investigated concentrations of salsolinol (50 and 100 µM) diminished this apoptotic marker, while its highest dose (500 µM) significantly potentiated the glutamic acid effect on caspase-3 activity (Fig. 2a). Analogous effects were observed for LDH release (Fig. 3a), which suggests that salsolinol has a clear biphasic effects; at lower concentrations (50 and 100 µM) it demonstrated the neuroprotective activity, whereas in the highest dose (500 µM), it caused neurotoxic effect. Additionally, in mouse striatum cultures, both investigated doses of salsolinol (50 and 500 µM) revealed the neuroprotective function (Figs. 2b, 3b). In contrast to the previous studies, we investigated for the first time whether salsolinol can destroy nondopaminergic structures, e.g., the hippocampus. It is composed of different kinds of neurons and contains a high proportion of glutamatergic and small amount of dopaminergic neurons. For this reason, it is a very sensitive structure to the glutamate-induced toxicity and is a good model for investigation of apoptosis.

Storch et al. (2000) concluded that salsolinol was toxic to dopaminergic neuroblastoma SH-SY5Y cells by blocking the cellular energy supply via inhibition of mitochondrial complex II activity. The latter authors found that incubation of human SH-SY5Y dopaminergic neuroblastoma cells with salsolinol resulted in a rapid, dose- and time-dependent decrease in the intracellular level of ATP and ATP/ADP ratio of intact cells (Wanpen et al. 2004). Other in vitro studies showed that salsolinol induced specific changes in cellular energy metabolism, similar to those caused by MPP^+^, which consistently preceded cell death (Storch et al. 2000). As Morikawa et al. (1998) reported, salsolinol inhibited mitochondrial complex II activity. It caused a rapid loss of intracellular ATP and maximal turnover of glycolysis without compensating for fast energy depletion. Additionally, the blockade of complex II did not change the level of NADH. Selective binding of salsolinol was confirmed not only in dopaminergic structures, such as the striatum, but also in the pituitary gland, cortex, and hypothalamus (Homicsko et al. 2003). In fact, our present in vitro experiments showed that salsolinol used in a low 50 µM concentration in primary hippocampal cultures did not affect the mitochondrial membrane potential; however, in the highest dose of 500 µM, like glutamate, it produced the loss of mitochondrial membrane potential. What is more, salsolinol applied together with glutamate did not antagonize its toxicity connected with the dysfunction of mitochondrial membrane (Fig. 6).

It is important to underline that our in vitro studies performed on hippocampal primary cultures of both mice and rats clearly indicate that neurotoxic action of the highest investigated concentration of salsolinol (500 µM) occurs via apoptosis. Beyond the verification of caspase-3 activity or LDH release, other markers of apoptosis, such as the identification of apoptotic cells by Hoechst 33342 and calcein AM staining in the rat hippocampal cultures, were assessed. We also observed a biphasic effect of salsolinol, depending on its concentration, in Hoechst 33342 and calcein AM staining analysis. Salsolinol at 50 µM normalized the number of healthy living cells and diminished the number of fragmented nuclei caused by glutamic acid (Fig. 4c). However, salsolinol at 500 µM elevated the glutamate-induced pro-apoptotic effect on hippocampal cells (Fig. 4d).

The latter findings suggest that salsolinol may regulate the function of dopamine neurons as a neurotransmitter and may act as a mediator in the dopamine system (Naoi et al. 2004). Salsolinol antagonized behavioral action of l-DOPA and apomorphine, a dopamine receptor agonist (Ginos and Doroski 1979; Antkiewicz-Michaluk et al. 2000). Binding studies demonstrated that salsolinol displaced [^3^H] apomorphine, but not dopamine D_1_ ([^3^H]SCH23,390) and D_2_ ([^3^H]spiperone) receptor antagonists, from their binding sites, with effectiveness comparable to that of dopamine (Antkiewicz-Michaluk et al. 2000). The above data suggest that salsolinol may suppress dopaminergic transmission by acting on the agonist binding sites of dopaminergic receptors, which are different from neuroleptic binding sites. Salsolinol showed antidopaminergic profile since it induced only a weak effect on spontaneous locomotor activity. Moreover, it efficiently antagonized behavioral and biochemical effects of apomorphine and induced muscle rigidity (Antkiewicz-Michaluk et al. 2000; Lorenc-Koci et al. 2000; Vetulani et al. 2001).

Our ex vivo studies provided further interesting information about the mechanism of salsolinol action depending on the concentration. The present biochemical analysis demonstrated that a single dose of salsolinol (100 mg/kg) produced no changes in the concentration of dopamine and its metabolites in different rat brain structures. Its chronic (14 consecutive days) administration did not produce any changes in dopamine concentration or in the level of its metabolites, as well. On the other hand, as was recognized so far, the administration of salsolinol jointly with l-DOPA enhanced its effect. In fact, the level of dopamine and all its metabolites was significantly higher compared to a group treated with l-DOPA alone (data not shown). Additionally, both acute and chronic (14 days) administrations of salsolinol (100 mg/kg i.p.) did not affect the level of α-synuclein and tyrosine hydroxylase measured with 3 or 24 h withdrawal (Figs. 7a, b, 8a, b). The results seem to be consistent with the previously demonstrated salsolinol physiological ability to be a potent stimulator of prolactin (PRL) release (Toth et al. 2002; Hashizume et al. 2008, 2010), especially during lactation (Homicsko et al. 2003; Misztal et al. 2010). The fact is that PRL secretion is under the dominant and tonic inhibitory control of DA, and there is no consensus about the nature and identity of physiologically relevant PRL-releasing factors. However, it has been demonstrated that salsolinol, a DA-derived compound, is a putative endogenous regulator of PRL release in rats. Salsolinol administration to freely moving rats dose-dependently increased plasma concentrations of PRL; in addition, stress- and suckling-induced release of PRL in rats was blocked by an antagonist of salsolinol (1MeDIQ). The studies of Hashizume et al. (2008, 2009) have shown that intravenous (i.v.) injection of salsolinol stimulates the release of PRL in adult female goats. Another research group has also demonstrated that salsolinol is present in the infundibular nucleus-median eminence in lactating sheep and that the extracellular concentration of this compound increases in response to a suckling stimulus, which is associated with an increase in plasma PRL concentrations (Misztal et al. 2008).

In summary, the presented study indeed indicates that salsolinol might exert the opposing effects in the brain depending on the applied concentration. It reveals neuroprotective activity in a low concentrations and pro-apoptotic effects in the higher one. However, this naturally occurring THIQ amine used in high concentration and upon prolonged exposure causes apoptotic cell death and can be one of the etiological factors of neurogenerative disease.
